# Sensorimotor Synchronization with Different Metrical Levels of Point-Light Dance Movements

**DOI:** 10.3389/fnhum.2016.00186

**Published:** 2016-04-27

**Authors:** Yi-Huang Su

**Affiliations:** Department of Movement Science, Faculty of Sport and Health Sciences, Technical University of MunichMunich, Germany

**Keywords:** visual rhythm, sensorimotor synchronization, dance, music, biological motion

## Abstract

Rhythm perception and synchronization have been extensively investigated in the auditory domain, as they underlie means of human communication such as music and speech. Although recent studies suggest comparable mechanisms for synchronizing with periodically moving visual objects, the extent to which it applies to ecologically relevant information, such as the rhythm of complex biological motion, remains unknown. The present study addressed this issue by linking rhythm of music and dance in the framework of action-perception coupling. As a previous study showed that observers perceived multiple metrical periodicities in dance movements that embodied this structure, the present study examined whether sensorimotor synchronization (SMS) to dance movements resembles what is known of auditory SMS. Participants watched a point-light figure performing two basic steps of Swing dance cyclically, in which the trunk bounced at every beat and the limbs moved at every second beat, forming two metrical periodicities. Participants tapped synchronously to the bounce of the trunk with or without the limbs moving in the stimuli (Experiment 1), or tapped synchronously to the leg movements with or without the trunk bouncing simultaneously (Experiment 2). Results showed that, while synchronization with the bounce (lower-level pulse) was not influenced by the presence or absence of limb movements (metrical accent), synchronization with the legs (beat) was improved by the presence of the bounce (metrical subdivision) across different movement types. The latter finding parallels the “subdivision benefit” often demonstrated in auditory tasks, suggesting common sensorimotor mechanisms for visual rhythms in dance and auditory rhythms in music.

## Introduction

Musical rhythms encompass multiple metrical levels of periodicity. While listeners can tune to different levels (each one termed a *pulse*) and hence different tempi in the same rhythm, each individual typically identifies a most salient periodicity as the *beat* (Drake et al., 2000; McKinney and Moelants, 2006). Perceptual grouping of alternating strongly and weakly accented events, or stronger and weaker beats, gives rise to the musical meter, which functions as a temporal reference frame and yields a distinct percept of patterning in music (London, 2012). These temporal modules not only define the structure of musical rhythms, but more importantly engage human behaviors. People move their bodies naturally to the musical beat, and the movements often consist of regular patterns (Toiviainen et al., 2010; Su and Pöppel, 2012; Manning and Schutz, 2013; Burger et al., 2014). This is an everyday example of coordinating one’s motor output with external sensory rhythms, known as sensorimotor synchronization (SMS; Repp and Su, 2013). Though better understood in humans, SMS in various forms—albeit to different degrees—has also been observed in other non-human animals (Fitch, 2013; Repp and Su, 2013). From an evolutionary point of view, synchronization behaviors serve to coordinate individuals with each other as well as in response to environmental signals. As SMS requires tracking the underlying periodicity of sensory rhythms (Merker et al., 2009), this process would be hindered if no temporal regularity can be perceived. Thus, the metrical structure of musical rhythm plays a functional role in SMS for humans, and this function may have evolutionary purposes shared by rhythmic behaviors in other species (Ravignani et al., 2014).

Most knowledge of rhythm perception and SMS originates from findings of auditory tasks for two possible reasons. For one, rhythm in human society is most readily ascribed to characteristics of auditory stimuli, such as music or speech, whose prevalent role in communication makes the research question relevant. For another, it seems unfeasible to manipulate rhythmic visual stimuli such that various metrical structures, e.g., different simultaneous periodicities, can be naturally presented as in the auditory tasks. As such, theoretical frameworks of rhythm processing, such as how the perceptual system entrains to the hierarchical periodicities, have mainly been developed and tested in the auditory domain (Large and Snyder, 2009; London, 2012). While the mechanism of temporal tracking has been postulated to be generalizable to the visual modality (Large and Jones, 1999), there has been little verification. The lack of investigation on visual synchronization overlooks the significance of rhythmic visual cues in guiding timed actions, which is supported by animal research: for example, monkeys exhibit similar temporal sensitivity to auditory and visual signals (Zarco et al., 2009); they also show signs of synchronization to ecologically-relevant visual rhythms, such as regular limb motions of another monkey (Nagasaka et al., 2013). Furthermore, visual cues as communicated by structured patterns of body movements (i.e., “dancelike” movements) are important signals to regulate inter-individual behaviors in the animal kingdom, e.g., in songbirds’ courting rituals (Ota et al., 2015), and possibly also in Chimpanzees’ playing (Oota, 2015). These behaviors give insight into how humans synchronize to visual cues derived from movement patterns, which manifest most evidently in dance (Kirsch and Cross, 2015) and may even be underpinned by overlapping mechanisms as synchronizing to music (Su, 2016).

When gauging human SMS of simple movements (finger tapping) with relatively simple auditory stimuli (isochronous tones), the metrical structure of auditory rhythm has been found to modulate SMS in various ways (Repp, 2005; Repp and Su, 2013). One repeatedly shown finding is that, within an inter-beat interval (IBI) of 200–1800 ms, synchronization to the beat is stabilized by the presence of metrical subdivisions in tasks known as “1:*n* tapping” (*n* = 2, 3, or 4, i.e., tapping to every second, third, or fourth event, Repp, 2003; Zendel et al., 2011; Madison, 2014). This effect, termed “subdivision benefit” (Repp, 2003), has also been demonstrated in subdivisions that are mentally imposed (Repp and Doggett, 2007; Repp, 2008a). That a parallel, lower-level periodicity—either physically or mentally imposed—can influence SMS with the beat indicates that rhythm perception and synchronization involve tracking multiple periodicities simultaneously, during which temporal information across several metrical levels may be integrated (Repp, 2008b). This idea is further supplemented by findings that the same beat tempo is perceived to be slower with than without the presence of metrical subdivisions (Repp, 2008a; Su, 2016), pointing to the effect of a lower-level pulse on temporal processing of an attended beat. It is less clear, though, whether the other way around is also true, i.e., whether SMS with a less salient pulse is modulated by the presence of a higher-level, more salient beat. An earlier study suggested that adding metrical accents may assist offbeat tapping to an otherwise unaccented isochronous sequence (Keller and Repp, 2005). Beyond that, there seems to be no systematic investigation in this regard.

Human SMS studies employing comparable auditory and visual stimuli (Repp, 2003; Patel et al., 2005; Lorås et al., 2012) often demonstrated inferior synchronization in the latter. The adopted visual stimuli were, however, rather simple (e.g., flashes) and bore little resemblance to the environmental signals. Recent studies started incorporating dynamic visual stimuli that move with realistic object or biological kinematics, such as a bouncing ball (Hove et al., 2013b; Iversen et al., 2015) or a bouncing human figure (Su, 2014b). Synchronization to such periodic movements improves considerably compared to situations of repetitive flashes, suggesting better visual SMS capacity than previously believed. Nevertheless, these visual rhythms contain only one periodicity, and the obstacle remains as to how to investigate SMS to visual stimuli with even more complex rhythmic structure. It is still unclear whether visual synchronization with realistically moving stimuli—especially if they contain rich metrical information—can engage similar mechanisms to what is known for auditory rhythms. For example, if multiple metrical periodicities were simultaneously present in the visual stimuli, would such a phenomenon as the “subdivision benefit” be observed?

**Figure 1:**
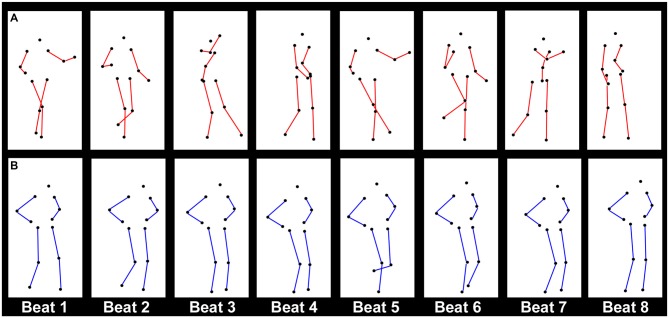
**Illustration of the point-light motion stimuli used in both experiments, showing one cycle of (A)** *Charleston* **dance and (B)** *Balboa* **dance in its natural version.** One cycle corresponds to eight metronome beats. The eight columns for each dance are taken from the frames in the stimuli representing the posture at each beat. In both examples, the figure moves with horizontal translational motion (TM). The sequences illustrate the limb movement patterns, while the regular bounce of the trunk is not immediately obvious here. For the purpose of visualization, the colors are inverted for the markers and the background, and lines connecting the joints (red for *Charleston* and blue for *Balboa*) are drawn here, which did not exist in the visual stimuli as displayed in the experiments.

**Figure 2:**
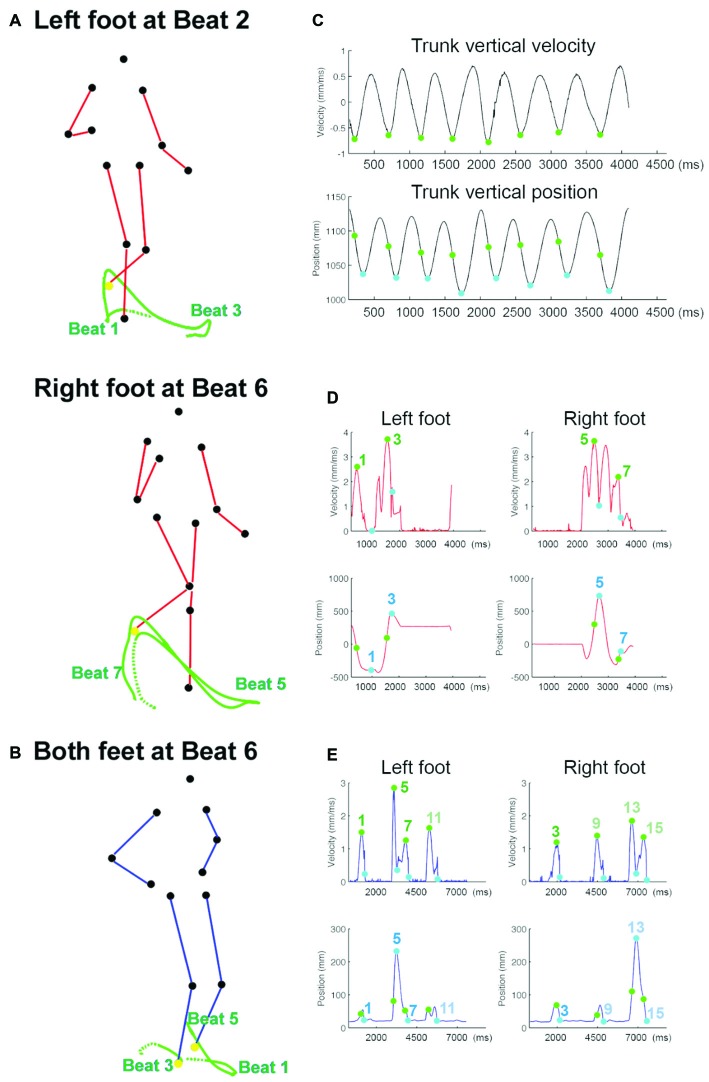
**The trajectory and kinematics of the point-light motion stimuli. (A)** The tracked trajectories, shown in green lines, of the left foot (upper panel) and the right foot (lower panel) in *Charleston*, plotted on the frame at Beat 2 and Beat 6, respectively. The left foot positions at Beat 1 and 3, and the right foot positions at Beat 5 and 7, are noted relative to each plotted trajectory. The tracked foot marker is shown in yellow in the respective panel. The dotted trace represents the trajectory leading up to the earliest beat in each panel. The two frames are taken from the same perspective relative to the PLF movement, and TM can be seen here as the PLF has moved forward in the lower panel relative to the upper one; without TM there is no such horizontal displacement. **(B)** The tracked trajectories (green lines) of the right and left foot (both in yellow) in *Balboa*, plotted on the frame at Beat 6. The trace between Beat 1 and Beat 5 belongs to the left foot, and the trace before Beat 3 belongs to the right foot. TM can also be seen here as the horizontal displacement of both feet relative to the starting position (the beginning of each dotted trace). **(C)** The velocity and position profile of the trunk movement, averaged across the four trunk markers along the time vector (*X*-axis). This profile is taken from one cycle of *Charleston* without TM at the IBI of 500 ms; the trunk kinematic pattern in other dance movement conditions is essentially the same. The green and blue circles mark the point of peak velocity and the point of lowest position in each bounce, respectively. **(D)** The 3D velocity (upper panel) and the Y (sagittal) position profile (lower panel) of the foot movement in one cycle of *Charleston*, IBI = 450 ms, with TM. The green and blue circles mark the point of peak velocity and the point of end position in each trajectory, with the corresponding beat number notated. **(E)** The 3D velocity (upper panel) and the vertical position profile (lower panel) of the foot movement in two cycles of *Balboa*, IBI = 450 ms, with TM. Points of peak velocity and end position are illustrated in the same manner as in **(D)**; beat 9–15 occur in the second cycle.

The present study addressed these issues using novel, naturalistic visual stimuli of a set of human dance movements developed in a recent study (Su, 2016). Given that the link between musical rhythms and human movements is well reflected in how humans move, or dance, to music (Burger et al., 2014), dance observation may perpetuate visual rhythm perception based on action-perception coupling. Specifically, as dance movements can embody the metrical structure of music (Naveda and Leman, 2010; Toiviainen et al., 2010), when presented as visual stimuli they may communicate multiple levels of periodicity in parallel, providing a suitable and ecological analog to auditory musical rhythms. The recent study (Su, 2016) presented stimuli of a point-light figure (PLF, Johansson, 1973) performing basic steps of *Charleston* and *Balboa* dance cyclically (Figure 1). An important characteristics of both dances was the regular bounce (generated by knee flexion and extension) that can be seen especially in the trunk movement pattern (Figure 2C). Besides, in *Charleston* the limbs moved with relative large trajectories in space (regular leg and arm swinging, Figure 2A), whereas in *Balboa* the legs moved in a footstep-like manner, and the arms remained still (Figure 2B). A critical feature in both dances was that the trunk bounced vertically at every beat while the limbs moved laterally at every second beat, yielding two possible metrical levels in the movements. It was found that observers could tune to either periodicity flexibly, with the leg movements more often perceived as beat than the bounce. Moreover, the tempo of the leg movements (beat) was perceived to be slower with than without the trunk bouncing simultaneously (subdivisions), mirroring previous auditory findings (Repp, 2008a). From here on, it seems logical to examine visual SMS with these stimuli as a next step, in an attempt to answer the questions raised above: namely, effects of different metrical structures in the movement on SMS.

In two finger-tapping tasks, the present study investigated two different effects of visual metrical structure on SMS with the point-light dance movements: synchronizing to the pulse with or without metrical accents (Experiment 1), and synchronizing to the beat with or without metrical subdivisions (Experiment 2). In Experiment 1, participants observed the PLF performing the two dance movements and tapped to the bounce of the trunk in a synchronized manner. For the *Charleston* stimuli, which involved the trunk bouncing and the legs and arms moving in a symmetrical manner, movement variations were implemented to examine whether tapping to the bounce (i.e., lower-level pulse) was stabilized by the presence of the leg movement, the arm movement, or both (i.e., higher-level metrical accents). For the *Balboa* stimuli, which involved only the trunk and the legs, the legs could move either in the same tempo as the bounce (i.e., accents at the same level as the pulse), or twice as slow as the bounce (i.e., accents at one metrical level higher than the pulse)^1^. Besides the effect of leg movement on tapping to the bounce, it was of interest whether this effect was modulated by the metrical relation between the two periodicities.

In Experiment 2, participants observed the same dancing PLF and tapped synchronously to the leg movements. Both dance movements were presented either naturally, or without the trunk bouncing in the stimuli. If visual SMS engages similar mechanisms as the auditory counterpart, tapping to the leg movements (i.e., beat) should be more stable with than without the simultaneous trunk movement (i.e., metrical subdivisions, Repp, 2003; Zendel et al., 2011; Madison, 2014). Moreover, the *Charleston* stimuli probed whether adding another metrical accent, i.e., the arm movement, would further stabilize synchronization. Finally, as an additional variable of interest, the PLF in both experiments danced either with horizontal translational motion (TM), i.e., the whole body moving forward and backward regularly (see Su, 2016), or with the whole body remaining in place (without TM). This was meant to examine whether effects of metrical accent or metrical subdivision were modulated by horizontal spatial information in the entire movement.

## Experiment 1: Synchronizing to the Bounce

### Methods

#### Participants

Eighteen young, healthy volunteers (five males, mean age 26.3 years, *SD* = 4.8) took part in this experiment. Participants were naïve of the purpose, gave written informed consent prior to the experiment, and received an honorarium of 8 € per hour for their participation. Participants were not pre-screened for musical or dance training, which ranged from 0 to 21 years (all amateurs). Thirteen and eight participants had trained in music and dance (but none in swing dance), respectively, amongst whom six had trained in both. The mean duration of music and dance training was 5.3 years (*SD* = 5.2) and 2.6 years (*SD* = 3.4). The study had been approved by the ethic commission of Technical University of Munich, and was conducted in accordance with the ethical standards of the 1964 Declaration of Helsinki.

#### Stimuli and Materials

The visual stimuli consisted of a human PLF performing basic steps of *Charleston* and *Balboa* dance in two different tempi. The stimuli had been generated by recording a swing dancer performing these steps using a 3-D motion capture system (Qualisys Oqus, 8 cameras at a sampling rate of 200 Hz, with 13 markers attached to the joints, Johansson, 1973) paced by metronomes with an IBI of 500 and 550 ms, respectively. The stimuli were a subset of the movement sequences used in a recent study (Su, 2016), where the stimuli preparation and construction were reported in detail. The description here will thus be brief.

Each dance was performed in continuous cycles, with one cycle corresponding temporally to eight metronome beats (Figure 1). In both dances, the trunk bounced vertically at every beat (beat 1–8), which was conveyed by movement patterns of the shoulder and the hip markers on both sides. The limbs (legs and arms for *Charleston*, and legs for *Balboa*) moved laterally at every second beat (beat 1, 3, 5, and 7), where the leg movements were conveyed by the knee and the foot markers on both sides, Figures 2A,B. The PLF performed these movements either with horizontal TM, or mostly in place (no TM, see Su, 2016). The best cycle amongst the recorded ones performed in a given condition (i.e., at a given tempo, with or without TM) was looped as visual stimuli. The *Charleston* dance presented here was authentic of the repertoire. The *Balboa* dance was presented both as in the original repertoire, wherein the legs moved at the same tempo as the trunk, and in a modified version, wherein the legs moved at half of the trunk tempo (see Su, 2016). The inclusion of both versions of the *Balboa* was meant to compare effects of leg movements that moved at the same metrical level as the bounce (the original version), or at one metrical level higher (the modified version).

The main manipulation was the presence or absence of the limb movements in parallel to the trunk bouncing. For *Charleston*, four movement variations were created: (1) the whole body moved naturally as recorded (termed “*Trunk + Arms + Legs*” to reflect the moving body parts); (2) the arm movements were removed by replacing the trajectories of the elbow and hand markers on both sides with similar ones as in *Balboa*, i.e., the palms were placed on the hips throughout (termed “*Trunk + Legs*”); (3) the leg movements were removed by replacing the trajectories of the knee and foot markers on both sides with a constant position on the *X* and *Z* dimension (taken from the first frame), while their *Y* positions (along the sagittal plane) were made to change in the same magnitude as the hip markers (termed “*Trunk + Arms*”); and (4) both the leg and the arm movements were removed by combining manipulations in (2) and (3), leaving only trunk movements intact (termed “*Trunk only*”). For *Balboa*, two variations were introduced: (1) natural as recorded (“*Trunk + Legs*”, as there was no arm movement in *Balboa*); and (2) leg trajectories removed in the same manner as described in *Charleston* (“*Trunk only*”). Note that all the manipulations were carried out on the first (natural) movement condition, and thus the trunk movement was identical across all conditions for each dance. Besides, in conditions where the leg movements were artificially removed, all the leg markers remained present; if the PLF moved with TM, the leg markers moved back and forth with the upper body (as if sliding on wheels), and thus the image of a humanlike figure was preserved throughout the sequence.

The 3-D motion data of each dance were presented as point-light display on a 2-D monitor, using routines of Psychophysics Toolbox version 3 (Brainard, 1997) running on Matlab^®^ R2012b (Mathworks). The function *moglDrawDots3D* allowed for depth perception in a 2-D display. The PLF was represented by 13 white discs against a black background, each of which subtended 0.4° of visual angle. The whole PLF subtended approximately 5° (width) and 12° (height) when viewed at 80 cm. The PLF was displayed facing the observers, in a configuration as if the observers were watching from 20° to the left of the PLF, which served to optimize depth perception of biological motion in a 2-D environment.

#### Procedure and Design

The stimuli and experimental program were controlled by a customized Matlab script and Psychtoolbox version 3 routines running on a Linux Ubuntu 14.04 Long Term Support (LTS) system. The visual stimuli were displayed on a 17-inch CRT monitor (Fujitsu X178 P117A) with a frame frequency of 100 Hz at a spatial resolution of 1024 × 768 pixels. Participants sat with a viewing distance of 80 cm. The finger taps were registered by a customized force transducer that was connected to the Linux computer via a data acquisition device (Measurement Computing^®^, USB-1608FS). Data were collected at 200 Hz, which was controlled and synchronized on a trial basis by the experimental program in Matlab. Participants wore closed studio headphones (AKG K271 MKII) to avoid potential auditory distraction.

Participants self-initiated each trial by pressing the space key. On each trial, a PLF was shown performing either a *Charleston* or a *Balboa* sequence cyclically in one of the two tempi, either with or without TM. For each Tempo × TM condition, there were four movement variations regarding the moving body parts for *Charleston* and two for *Balboa* (as described in “Stimuli and Materials” Section). Participants’ task was to observe the PLF movement as a whole and tap to the bounce of the trunk in a synchronized manner. They tapped with the index finger of their dominant hand on the force transducer. In total six complete movement cycles were presented on each trial, equaling 48 bounces.

The experiment consisted of the following conditions: 4 (moving part) × 2 (TM) × 2 (tempo) for *Charleston*, and 2 (movement version: original or modified) × 2 (moving part) × 2 (TM) × 2 (tempo) for *Balboa*. All the conditions were presented in six blocks of 36 trials each, with all the conditions balanced across blocks and the order of conditions randomized within a block. Participants underwent six practice trials before starting the experiment. The entire experiment lasted around 2 h, completed in two sessions of three blocks each either on different days or on the same day with a longer pause (at least half an hour) in between.

#### Data Analysis

The timing of each tap was extracted by identifying the time point right before the amplitude of the measured force data exceeded a predefined threshold. The tap times were temporally aligned to the start of the visual stimulus, allowing for calculation of absolute asynchronies between each tap and the corresponding visual signal. The stimulus onset time, i.e., the beat as communicated by each bounce, was derived from the kinematic profile of the four trunk markers (shoulders and hips on both sides) averaged along the time vector for each sequence. Specifically, visual beat in a periodic biological motion may be communicated by both the position and the velocity parameters, such as the recurrent lowest position or the recurrent peak velocity of the bounce (Su, 2014a). Although several studies support the role of velocity cues (Luck and Sloboda, 2009; Wöllner et al., 2012; Su, 2014a), the position information might still influence where the beat was perceived (Su, 2014a; Booth and Elliott, 2015). As such, two sets of stimulus beat onset times were extracted, one based on the peak vertical velocity (termed “velocity beat”) and the other based on the vertical end position (termed “position beat”) of the bounce, with the former preceding the latter in every bounce (Figure 2C). Tap times were first calculated relative to each beat parameter separately. The first two taps in each trial were discarded from analyses.

The main index of synchronization was the stability of the taps relative to the beats (Repp and Su, 2013). As visual synchronization is known to be variable and the present stimuli were complex, circular statistics (Berens, 2009) was applied to analyze the tap-beat phase relations (Hove et al., 2013b; Iversen et al., 2015; see also Hove et al., 2012, for circular statistics applied to SMS with non-isochronous beats). Each tap time was converted to the phase relative to its closest beat on a circular scale (0–360° between two consecutive beats). For a given trial, the tap-beat stability was indexed by *R*, the mean resultant length of the relative phase vector. *R* ranged from 0 (taps distributed uniformly around beat onsets, suggesting no synchronization) to 1 (perfect synchronization with taps distributed unimodally relative to beat onsets, see also Kirschner and Tomasello, 2009, for a comprehensive description). The mean direction of the relative phase, *θ*, was also calculated for each trial, indexing the mean magnitude and direction of the tap-beat asynchronies. Both *R* and *θ* were first analyzed with respect to the velocity beat and the position beat separately.

### Results

Analyses were carried out for the two dances separately to answer different questions. For *Charleston*, of interest was the effect of leg movement, arm movement, or both, on synchronization with the bounce. For *Balboa*, it was of interest whether the effect of leg movement differed when the legs moved at the same tempo or at half the tempo of the bounce. All the analysis of variances (ANOVAs) reported in this study were repeated-measures ANOVA.

It should be noted that the different TM × tempo stimuli were generated by recording these movements performed separately in the respective condition, and the trajectory of each marker was not further spatially or temporally adjusted (in order to present authentic biological motion stimuli). There were thus inevitable differences in deviation from isochrony, as well as variations of kinematics, across different conditions. As such, results of TM and tempo will be focused on whether they interact with the main variable of interest, the moving part, in order to verify whether the effect of moving part generalizes to different movement conditions. In case of main effects of TM and tempo, or interactions between the two, the results will not be further discussed if they may be attributed to differences in the variability of beat timing (i.e., higher or lower tapping variability associated with higher or lower variability of the beat onset times). The same rules will apply to results of Experiment 2.

#### Determining the Synchronization Target

First, in order to identify which kinematic feature participants synchronized to, the individual means of angular direction (*θ*) were analyzed in a full factorial ANOVA for *Charleston*: 4 (moving part) × 2 (TM) × 2 (tempo) × 2 (beat parameter: velocity or position), and for *Balboa*: 2 (movement version) × 2 (moving part) × 2 (TM) × 2 (tempo) × 2 (beat parameter). In both ANOVAs, there was a main effect of beat parameter, *F*_(1,17)_ = 27.26, *p* < 0.001, $\eta_{p}^{2}$ = 0.62, and *F*_(1,17)_ = 89.36, *p* < 0.001, $\eta_{p}^{2}$ = 0.84, both showing that taps were closer (less negative *θ*) to the velocity than to the position beat. For the *Charleston* stimuli, mean *θ* was −21.07° and −69.75° for the velocity and the position beat, respectively. For the *Balboa* stimuli, mean *θ* was −24.17° and −82.71°, respectively. Given that synchronization stability (*R*) was comparable with respect to the velocity and the position beat (both beat parameters yielded mean *R* = 0.70 for *Charleston*; both parameters yielded mean *R* = 0.75 for *Balboa*), the smaller magnitude of asynchrony was taken as evidence that the velocity beat was the preferred synchronization target in this experiment. The subsequent analyses were conducted on *R* with respect to the velocity beat.

#### Synchronization to the Beat

**Figure 3:**
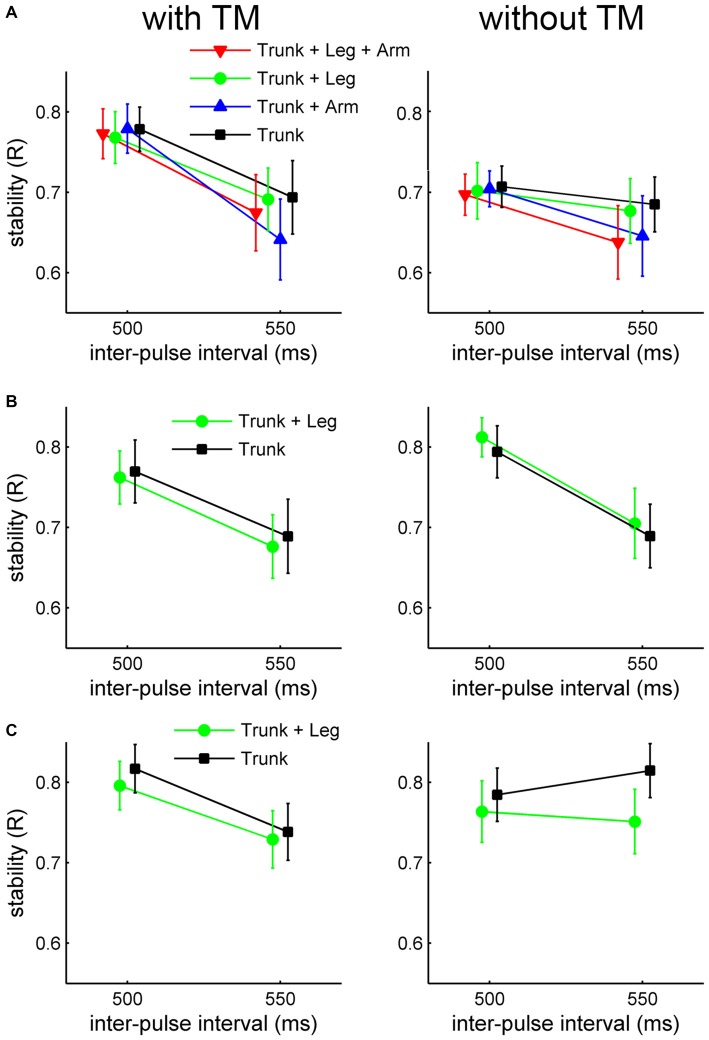
**Results of mean synchronization stability as indexed by *R* in Experiment 1. (A)** *Charleston* dance, **(B)** *Balboa* dance with the foot tempo the same as the trunk tempo, and **(C)** *Balboa* dance with the foot tempo twice as slow as the trunk tempo. *R* values are plotted as a function of tempo, for each moving part and TM condition separately. Error bars are standard error of the means.

For *Charleston*, the individual means of *R* were submitted to a 4 (moving part) × 2 (TM) × 2 (tempo) ANOVA. Moving part had no significant effect on *R*, *F*_(3,51)_ = 1.30, *p* > 0.2, $\eta_{p}^{2}$ = 0.07, nor interaction with any other variable. The main effect of tempo was significant, *F*_(1,17)_ = 9.71, *p* < 0.01, $\eta_{p}^{2}$ = 0.36, showing more stable synchronization for the faster tempo (IBI = 500 ms). The main effect of TM was also significant, *F*_(1,17)_ = 25.16, *p* < 0.001, $\eta_{p}^{2}$ = 0.60, showing greater stability for synchronizing to movements with TM than without (Figure 3A). There was a significant TM × tempo interaction, *F*_(1,17)_ = 7.44, *p* < 0.02, $\eta_{p}^{2}$ = 0.30; follow-up one-way ANOVAs showed that the effect of TM was only significant for IBI = 500, *F*_(1,17)_ = 40.29, *p* < 0.001, $\eta_{p}^{2}$ = 0.70, and not for IBI = 550, *p* > 0.3. The main effect of TM as well as its interaction with tempo could, however, be due to the corresponding variability of the stimulus beat.

For *Balboa*, the individual means of *R* were submitted to a 2 (movement version) × 2 (moving part) × 2 (TM) × 2 (tempo) ANOVA. Again, the main effect of moving part was not significant, *F*_(1,17)_ = 1.62, *p* > 0.2, $\eta_{p}^{2}$ = 0.09, and nor did it interact with other variables. Significant main effects were found for movement version, *F*_(1,17)_ = 8.24, *p* < 0.02, $\eta_{p}^{2}$ = 0.33 (greater *R* when the legs moved at half the bounce tempo), for TM, *F*_(1,17)_ = 6.28, *p* < 0.03, $\eta_{p}^{2}$ = 0.27 (greater *R* for movements without TM than with), and for tempo, *F*_(1,17)_ = 17.35, *p* < 0.001, $\eta_{p}^{2}$ = 0.51 (greater *R* for movement at the faster tempo), Figures 3B,C. These patterns were, however, in the same direction as the difference in stimulus beat variability between the respective conditions.

In sum, stability of synchronizing to the bounce was not affected by the presence of lateral limb movements, which was true whether the limbs moved at the same metrical level as the bounce, or at one level higher. For the *Charleston* stimuli, taps were more synchronized to the bounce at the faster tempo (nominal IBI = 500 ms). As an additional note, taps generally preceded the beat, which is reminiscent of the negative mean asynchronies (NMA) typically found in SMS with auditory stimuli (Repp and Su, 2013).

## Experiment 2: Synchronizing to the Leg Movements

This experiment examined whether synchronization to the beat was improved by the presence of metrical subdivisions. Participants tapped synchronously to the leg movements (beat) of both dances, in which the trunk bounced simultaneously (subdivisions) or not in the stimuli. While referred to as “beat” borrowing the musical terminology, the leg movements naturally deviated more from isochrony than the trunk bouncing. Nevertheless, observers were able to perceive and synchronize with the rhythm of these movements (Su, 2016).

### Methods

#### Participants

Twenty volunteers (nine male, mean age 25.3 years, *SD* = 5.2) took part in this experiment. Fourteen and 10 participants had trained in music and dance, respectively, amongst whom six had trained in both. The music and dance training duration ranged from 0 to 16 years (all amateurs), with a mean of 5.1 years (*SD* = 4.3) and 3.6 years (*SD* = 5.0), respectively. The participant handling and ethics procedure was the same as in Experiment 1.

#### Stimuli and Materials

The visual stimuli consisted of the same PLF performing *Charleston* and *Balboa* dance, with and without TM, in two different tempi corresponding to the metronome IBI of 400 and 450 ms (indicating the trunk tempi). The two tempi were chosen, as the previous study suggested that the leg movements and the bounce could be most optimally perceived in parallel in these tempi (Su, 2016). For *Balboa*, only the modified version was included, in which the legs moved at half of the bounce tempo. In both dances, the legs thus moved at an interval of around 800 and 900 ms, respectively. For consistency purpose, the tempo of the movement will still be referred to by the metronome IBI, 400 and 450 ms.

The main manipulation here was the presence or absence of the trunk bouncing simultaneously to the leg movements. For *Charleston*, three movement variations were included: (1) natural as it was (“Arms + *Trunk + Legs*”); (2) the arm movements were removed in the same manner as described in Experiment 1 (“*Trunk + Legs*”); and (3) both the arm and the trunk movements were removed (“*Legs only*”), in which the trunk bounce was removed by keeping constant the vertical position of the shoulder and hip markers, while leaving their positions in the horizontal plane as natural (see Su, 2016, Experiment 2). For *Balboa*, two variations were included: (1) natural as it was (“*Trunk + Legs*”); and (2) with the trunk bounce removed (“*Legs only*”).

#### Procedure and Design

The setup and the procedure were the same as in Experiment 1. The task was now to tap to the leg movements in a synchronized manner. The experimenter made sure every participant understood the pattern of leg movements they should tap to. Eight complete movement cycles were presented in each trial, equaling 32 leg movements.

In total the following conditions were included: 3 (moving part) × 2 (TM) × 2 (tempo) for *Charleston*, and 2 (moving part) × 2 (TM) × 2 (tempo) for *Balboa*. All the conditions were presented in eight blocks of 20 trials each, with the conditions balanced across blocks and the order of conditions randomized within a block. Participants underwent six practice trials before starting the experiment. The entire experiment lasted around 2 h, completed in two sessions of four blocks each.

#### Data Analysis

The visual beat was first defined by the peak velocity and the end position of the foot markers separately. The velocity beat was calculated as the time point of the peak 3D (absolute) velocity in each leg trajectory. The position beat was defined by the time point of the end position in the *Y* (sagittal) and in the *Z* (vertical) dimension for each trajectory in *Charleston* and *Balboa*, respectively (Figures 2D,E). The velocity beat always occurred prior to the position beat. The tap times, synchronization stability (*R*), and mean relative phase (*θ*) were analyzed in the same manner as in Experiment 1.

### Results

#### Determining the Synchronization Target

To examine which kinematic feature served the synchronization target, the individual means of *θ* were first analyzed in a full factorial ANOVA, excluding the “*Trunk* + *Arms* + *Legs*” condition in *Charleston*: 2 (dance style) × 2 (moving part) × 2 (TM) × 2 (tempo) × 2 (beat parameter: velocity or position), which yielded a main effect of beat parameter, *F*_(1,19)_ = 12455, *p* < 0.001, $\eta_{p}^{2}$ = 0.99. It was found that taps lagged the velocity beat (mean *θ* = 51.49°) while leading the position beat (mean *θ* = −22.92°), suggesting that both parameters might have been taken into account for synchronization. In addition, the full ANOVA with the same factors was conducted on individual means of *R*, which revealed a significant interaction between beat parameter and dance style, *F*_(1,19)_ = 1558, *p* < 0.001, $\eta_{p}^{2}$ = 0.98. Partial ANOVAs showed that synchronization with *Charleston* stimuli was better in terms of the velocity beat, *F*_(1,19)_ = 59.99, *p* < 0.001, $\eta_{p}^{2}$ = 0.76 (*R* = 0.81 for velocity and *R* = 0.75 for position beat), whereas synchronization with *Balboa* stimuli was better in terms of the position beat, *F*_(1,19)_ = 811.4, *p* < 0.001, $\eta_{p}^{2}$ = 0.98 (*R* = 0.65 for velocity and *R* = 0.85 for position beat). As such, it was assumed that the velocity and the position beat served the more effective synchronization target for the *Charleston* and the *Balboa* stimuli, respectively. *R* values for each dance in the subsequent analyses were calculated according to the respective synchronization target.

See Table 1 for an overview of the timing parameters of the stimulus beat for each movement condition, as well as the observed mean *R*. Synchronization stability generally agreed with the variability of beat onset times, i.e., more regular velocity-defined beat for the *Charleston* stimuli and more regular position-defined beat for the *Balboa* stimuli.

**Table 1:** **Timing parameters of the stimulus beat (leg movements) in each movement condition, and the corresponding synchronization stability measured in Experiment 2**.

	Metronome IBI (ms)	Mean IBI (ms)	CV (%)	Mean Abs. Dev. (%)	*R*
*Charleston*	
Velocity beat	400	
	TM	782.58	10.7	5.0	0.83
	no TM	801.29	18.7	11.2	0.73
	450	
	TM	932.10	14.2	11.0	0.86
	noTM	912.26	12.3	8.9	0.81
Position beat	400	
	TM	771.29	19.8	6.5	0.74
	no TM	784.03	26.9	13.5	0.76
	450	
	TM	917.10	23.3	10.0	0.73
	no TM	901.29	19.2	13.1	0.78
*Balboa*	
Velocity beat	400	
	TM	790.16	34.2	28.7	0.56
	no TM	795.81	18.9	11.8	0.72
	450	
	TM	903.87	26.3	22.8	0.61
	no TM	888.06	21.4	14.7	0.71
Position beat	400	
	TM	798.06	12.4	10.5	0.86
	no TM	807.42	16.8	13.6	0.83
	450	
	TM	904.03	17.9	15.2	0.84
	no TM	903.06	14.4	12.2	0.86

*Mean IBI: the mean inter-beat interval calculated from the leg movements of each condition. CV: the variability of the beat onset times indexed by the coefficient of variations of the IBIs. Mean Abs. Dev.: the mean absolute deviation of the IBIs from twice the metronome interval. R: the mean synchronization stability across participants for each condition, pulling together conditions with and without trunk movement*.

#### Synchronization to the Beat

**Figure 4:**
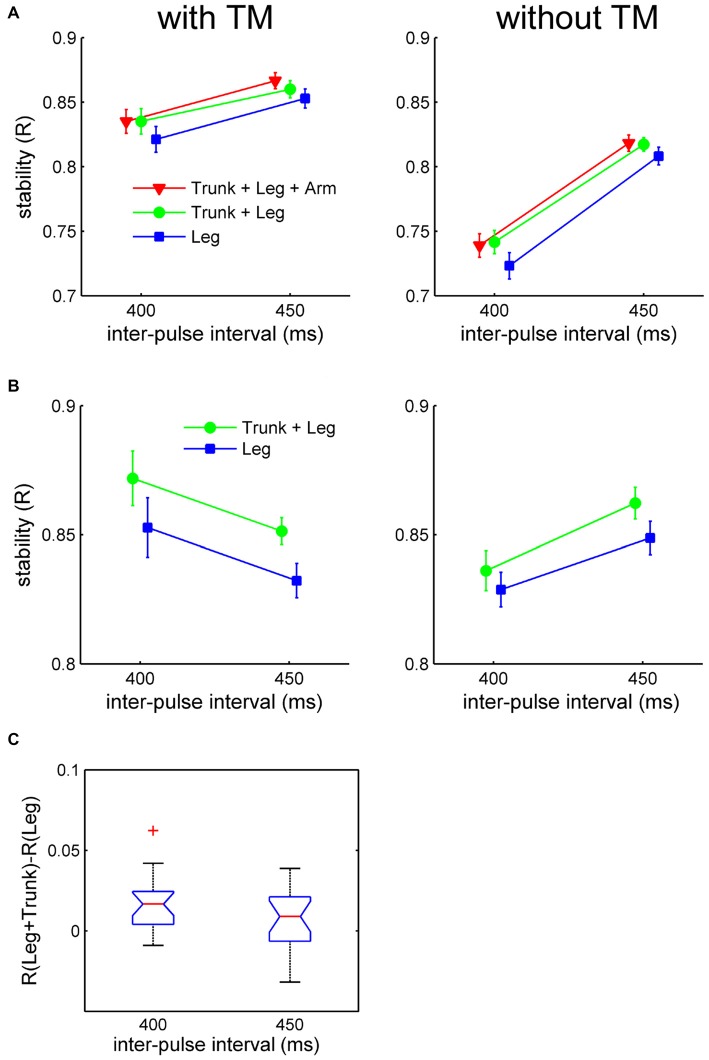
**Results of synchronization stability as indexed by *R* in Experiment 2. (A)** Mean *R* (with respect to the velocity beat) for *Charleston* dance, and **(B)** mean *R* (with respect to the position beat) for *Balboa* dance, plotted as a function of tempo for each moving part and TM condition separately. Error bars are standard error of the means. **(C)** Effect of moving part (“*Trunk + Legs*” vs. “*Legs only*”) contrasted at each tempo for *Charleston* dance, showing the mean difference score (the red horizontal line) with 95% CI (the blue notch). The whisker represents 99% of the data distribution.

The individual means of *R* were submitted to a 2 (dance style) × 2 (moving part) × 2 (TM) × 2 (tempo) ANOVA, not including the “*Trunk + Arms + Legs*” condition in *Charleston*. All four main effects were significant: (1) dance style, *F*_(1,19)_ = 62.27, *p* < 0.001, $\eta_{p}^{2}$ = 0.77, showing more stable synchronization with *Balboa* than with* Charleston*; (2) moving part, *F*_(1,19)_ = 29.83, *p* < 0.001, $\eta_{p}^{2}$ = 0.61, showing more stable synchronization to the leg movement with than without the simultaneous trunk movement; (3) TM, *F*_(1,19)_ = 234.3, *p* < 0.001, $\eta_{p}^{2}$ = 0.92, showing more stable synchronization to movements with TM; and (4) tempo, *F*_(1,19)_ = 34.73, *p* < 0.001, $\eta_{p}^{2}$ = 0.65, showing more stable synchronization to the slower movement tempo (IBI = 450 ms; Figures 4A,B). The effect of TM and Tempo may be associated with the variability of the stimulus beat.

Moving part was involved in a significant three-way interaction: dance style × moving part × tempo, *F*_(1,19)_ = 5.69, *p* = 0.027, $\eta_{p}^{2}$ = 0.23. Follow-up partial ANOVAs revealed that the moving part × tempo interaction was only (about) significant for *Charleston*, *F*_(1,19)_ = 4.77, *p* = 0.042, $\eta_{p}^{2}$ = 0.20, but not for *Balboa*, *F*_(1,19)_ = 0.76, *p* = 0.39, $\eta_{p}^{2}$ = 0.04. *Post hoc* one-way ANOVAs conducted for each tempo in the *Charleston* conditions showed better synchronization in the presence of the bounce at the faster tempo (IBI = 400 ms), *F*_(1,19)_ = 17.99, *p* < 0.001, $\eta_{p}^{2}$ = 0.49, but only marginally so at the slower tempo (IBI = 450 ms), *F*_(1,19)_ = 3.99, *p* = 0.06, $\eta_{p}^{2}$ = 0.17.

The interaction in *Charleston* was further confirmed by contrasting the effect of moving part for each tempo, using 95% confidence intervals (CI) of the difference scores across participants (Masson and Loftus, 2003; Cumming, 2014), i.e., difference in *R* between conditions with and without trunk for each tempo in *Charleston*. As shown in Figure 4C, only for IBI = 400 ms was the difference between conditions greater than zero at the 95% CI.

**Figure 5:**
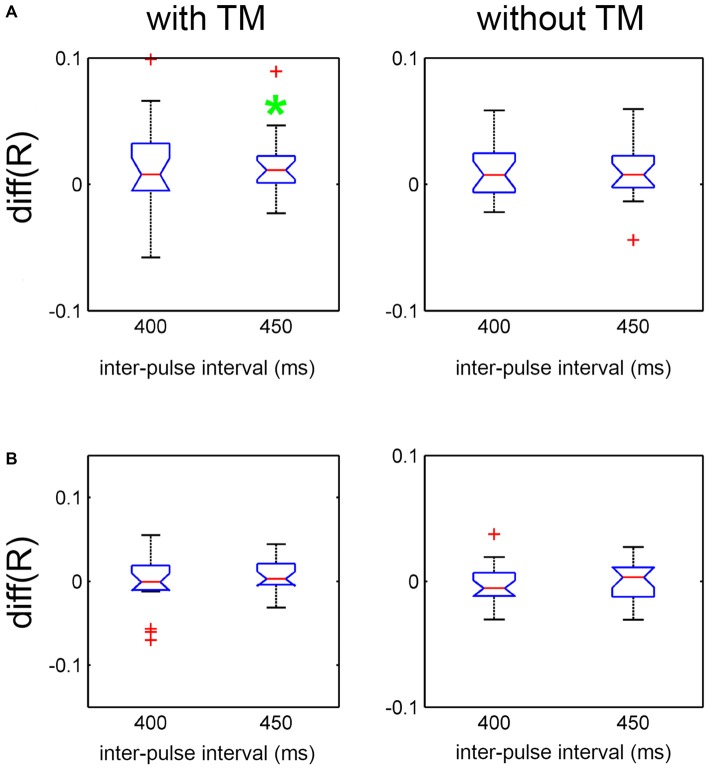
**Effect of moving part on *R* contrasted at each tempo and TM condition for *Charleston* dance in Experiment 2. (A)** Effect indexed by the difference in *R* between “*Arms + Trunk + Legs*” and “*Legs only*”. **(B)** Effect indexed by the difference in *R* between “*Arms + Trunk + Legs*” and “*Trunk* + *Legs*”. The red line and the blue notch represent the mean difference score and its 95% CI, respectively. The whisker represents 99% of the data distribution, and data points outside this range are plotted as red crosses. The green asterisk denotes the condition with a significant difference at 95% CI.

Finally, to examine the effect of the presence of arm movement compared to the other two moving part conditions in *Charleston*, difference scores were computed between *Arms + Trunk + Legs* and *Legs only*, by subtracting the latter from the former (Figure 5A), as well as between *Arms + Trunk + Legs* and *Trunk + Legs* (Figure 5B). The effects as indexed by the 95% CI of the difference scores were contrasted for each tempo and TM condition separately. As shown, synchronization was better with *Arms + Trunk + Legs* compared to *Legs only* at the slower tempo (IBI = 450 ms) with TM. None of the other comparisons showed an effect.

In summary, across all movement variations, synchronizing to the leg movement was more stable when the trunk was bouncing simultaneously at twice the leg tempo. For the *Charleston* stimuli, the effect of trunk movement was more evident at the faster tempo (IBI = 400 ms). Between the two dances, the velocity beat served a more effective synchronization target for *Charleston*, whereas the position beat was more effective for *Balboa*. Lastly, while the presence of trunk movement assisted tapping to the leg movement, adding another lateral limb movement (the arms) around the same tempo as the legs did not further improve synchronization.

## Discussion

The present study investigated visual SMS with biological motion stimuli of a dancing PLF. The dance movements were such that two metrical levels of periodicity were visually available, with the lateral leg movements being twice as slow as the vertical trunk bouncing, and the former more often perceived as beat (Su, 2016). To verify whether synchronization to metrical visual stimuli resembles that to auditory rhythms, two tapping experiments examined effects of metrical accent (leg movement) on synchronization to the pulse (bounce), as well as effects of metrical subdivision (bounce) on synchronization to the beat (leg movement). The main results show that, while metrical accents did not influence synchronization to a lower-level pulse, metrical subdivisions improved synchronization to the beat compared to the absence thereof. The latter finding replicated the subdivision benefit consistently shown in SMS with auditory rhythms (Repp, 2003; Zendel et al., 2011; Madison, 2014).

That the subdivision benefit was observed using visual dance stimuli has at least three theoretical implications. First, it extends a well-established auditory finding to the visual modality, suggesting similar rhythm processing across the two senses (Hove et al., 2013a). In auditory SMS, this effect has mainly been shown in metronomic stimuli, but not as consistently replicated in real music (Martens, 2011). One possible reason is that the rich metrical structure in music, perhaps strengthened by other cues such as pitch or melodic contour (Lerdahl and Jackendoff, 1983), may have led to a ceiling effect of SMS to the beat. The present result generalizes the subdivision effect to realistic visual stimuli across different dance styles and movement variations. At the same time, the effect can be argued to reside within the visual modality, as it seems unlikely that such complex stimuli would be recoded into auditory representation to guide behaviors (Guttman et al., 2005; Grahn et al., 2011). Secondly, building on recent findings of visual SMS with a single motion periodicity (Hove et al., 2013b; Su, 2014b; Iversen et al., 2015), the present biological stimuli contained multiple periodicities, making this effect not only ecologically plausible in the visual domain, but also comparable to music. Visual rhythms can thus be defined beyond simple stimuli to mirror their auditory counterpart. While both musical rhythms and the trajectories of dance movement often deviate from isochrony, in both cases the listeners and observers are able to extract the underlying regularity and track hierarchical levels of periodicity simultaneously, which in turn modulates motor behaviors (Large and Palmer, 2002; Large et al., 2002). This supports the idea that similar sensorimotor mechanisms may underlie auditory synchronization to music and visual synchronization to dance. Finally, regarding rhythm in the action-perception framework (Prinz, 1997; Maes et al., 2014), the subdivision benefit confirms how the metrical structure is visually perceived in dance movements that embody this structure (Su, 2016). This in turn suggests that rhythm perception, which is a prerequisite for SMS (Repp and Su, 2013), can be evoked by temporally structured auditory stimuli, as well as visual information of movements performed in response to these auditory rhythms. As both can engage the motor system (Schubotz, 2007; Hove et al., 2013a), observing rhythmic movements being arguably a form of motor simulation (Kirsch and Cross, 2015), the auditory rhythm of music and the visual rhythm of dance may indeed share a common sensorimotor representation.

In auditory musical rhythm as well as visual rhythm of biological motion, metrical subdivisions appear to facilitate synchronization by providing additional temporal information for the upcoming, attended beat (Madison, 2014). In the auditory stimuli, the effect can be explained by means of predictive temporal tracking (Large and Jones, 1999; Repp, 2008b). In dynamic visual stimuli as the present ones, it may involve the kinematics of one body part (the trunk) predicting that of another (the legs). The action-observation literature proposes that human observers form internal representation of familiar movement kinematics, which allows them to predict the spatiotemporal course of an action (Stadler et al., 2012). The predictive mechanism seems to apply to the action as a whole, rather than forming separate expectations for different body parts. As such, the kinematic information of all concurrently moving parts in a dance movement might be (automatically) integrated to make the eventual prediction of each “beat”. In this light, the bounce not only serves a finer temporal scale, but also provides additional kinematic cues for the leg movements. While it is beyond the current scope to elaborate on how the kinematic cues were visually integrated across different moving parts, it is worth noting that the obtained subdivision benefit could *not* have been confounded with participants tapping to the trunk movement when it was present. As the beat onset times of the trunk and of the legs did not coincide with each other, nor maintain a constant phase difference (see Table S1 in the “Supplementary Material” for a summary of these parameters), synchronization stability analyzed with respect to the leg movements would not have benefitted from taps synchronized to the trunk.

The other question asked in this study, i.e., whether tapping to the pulse would be stabilized by the metrical accent, was met with a negative answer. There has been little research in the auditory (and none in the visual) domain to address this issue, except for one study on offbeat tapping (Keller and Repp, 2005). The present result suggests that, visually, imposing an additional metrical frame yields no more gain on temporal coordination than what the lower-level periodicity already entails. The same might be speculated for the auditory stimuli. While the brain does respond differentially to subjectively accented and unaccented events in an isochronous auditory sequence (Iversen et al., 2009; Potter et al., 2009; Fujioka et al., 2010), there is thus far no evidence that enhanced anticipation at the metrically accented level leads to overall better motor synchronization to the lower-level pulse. One possible reason applicable to both modalities is that the metrical accent yields alternating on-beat and off-beat positions, and the effect of one level may cancel out that of the other (Repp et al., 2008). Notably, the null result of metrical accent on SMS also applies to adding accents at the same level as the pulse (i.e., the original version *Balboa* in Experiment 1), as well as superimposing another accent at the same level as the existing one (i.e., adding arm movement along with leg movement in *Charleston* in Experiment 2). As such, it seems an additional metrical periodicity has a functional impact on SMS only when it subdivides the target IBI (Madison, 2014).

Horizontal TM in the dance movement did not modulate the effect of metrical accent or metrical subdivision on SMS. This was more surprising regarding the subdivision effect for *Balboa* dance. As the leg movements in *Balboa* did not consist of large lateral trajectories, one would expect that the horizontal spatial frame imposed by TM (such that the leg trajectories were additionally marked by the regular positions on the ground) would be necessary to induce visual metrical accent. The results suggest that, regardless of the magnitude of their trajectories, the leg movements were readily differentiated from the trunk bouncing as being more accentuating^2^. This pattern is consistent with our recent work (Su and Salazar-López, in press), showing that regular leg movements in the Flamenco dance repertoire are perceptually prioritized as visual beat over other moving body parts. Results here thus extend this finding to different movements and dance genres. The role of leg movements in visual beat perception and synchronization is reminiscent of the finding that the preferred tempo in musical rhythms (Moelants, 2002) corresponds roughly to that in locomotion (MacDougall and Moore, 2005). In both cases, the perceptual preference of beat seems to be linked to the motor representation of the lower limbs. Evolutionarily, this finding suggests that the functional purpose of rhythmic sounds may be at least in part associated with rhythmic patterns of locomotion, or other movements generated by the legs, which is critical for survival. Depending on the affinity to different sensory modalities, in some species these cues may also be extracted via movement observation (Nagasaka et al., 2013; Kirsch and Cross, 2015).

Finally, a few other effects are worth brief discussions. First, in Experiment 2 the subdivision benefit in the *Charleston* stimuli was more evident for the faster movement tempo (IBI around 400 ms for the bounce and 800 ms for the legs). Interestingly, in the recent study employing the same stimuli, the effect of subdivision (bounce) on slowing tempo perception of the leg movement was also more obvious at this tempo (Su, 2016). There might be a range of tempi in which the two metrical levels of the present movements—embodied by different body parts—can be most optimally perceived in parallel. Similarly, synchronization to the bounce of *Charleston* dance was more stable with the IBI around 500 ms than 550 ms (Experiment 1). Future research may investigate how different movement types performed by different body parts yield the optimal tempo for visual rhythm perception and SMS. Another notable result is that the synchronization target may be served by different kinematic parameters between different kinds of movements. As observed, while both velocity and position cues might influence visual beat perception (Su, 2014a), the velocity cues are more stable and thus more useful when synchronizing with movements of large lateral trajectories, such as the leg movement of *Charleston*. Position cues, on the other hand, may convey the more regular beat when the movement amplitudes are smaller and the trajectories more concentrated on one dimension (vertical in the legs of *Balboa*).

In conclusion, the present study demonstrates that SMS with visual rhythms of dance resembles SMS with auditory rhythms of music, in that metrical subdivisions benefit synchronization to the beat. Synchronization to the pulse, on the other hand, is not further improved by a higher-level metrical accent. While biological motion yields spatiotemporally complex visual signals, which may not be as precise as a metronome, rhythmic movements as in dance can embody the metrical structure in a comparable manner as music, which by observation modulates synchronization behaviors. The present results not only highlight the similarity in rhythm processing between the two sensory modalities, but more importantly link rhythm cognition of music and dance in a common framework of action-perception coupling.

## Author Contributions

Y-HS conceptualized and designed the study, prepared the stimuli, conducted the experiments (data collection carried out by the research assistant), analyzed the data, interpreted the results, and wrote the manuscript.

## Funding

This work and the author were supported by a grant from the German Research Foundation (DFG), SU 782/1–2. The publication of this work was supported by the German Research Foundation (DFG) and the Technical University of Munich (TUM) in the framework of the Open Access Publishing Program.

## Conflict of Interest Statement

The author declares that the research was conducted in the absence of any commercial or financial relationships that could be construed as a potential conflict of interest.

## Footnotes

^1^The variation of legs moving at the same tempo as the bounce was not possible for the *Charleston* dance due to biomechanical constraint in the movement, and was thus only implemented for *Balboa*.

^2^This effect was not likely contingent upon different amount of motion between the legs and the trunk. Within a cycle of *Charleston*, the total traveled distance of the legs (*M* = 382.35 cm) was greater than that of the trunk (*M* = 236.90 cm), whereas in *Balboa* the traveled distance of the trunk (*M* = 196.36 cm) was greater than the legs (*M* = 163.17 cm).
