# Supplementary material for: Sensorimotor Synchronization with Different Metrical Levels of Point-Light Dance Movements
Source: Front Hum Neurosci. 2016 Apr 27;10:186. doi: 10.3389/fnhum.2016.00186 (PMC4846664; doi:10.3389/fnhum.2016.00186)
Supplement: Supplementary file 1 [file Table_1.docx]

Supplementary Material

Sensorimotor synchronization with different metrical levels of point-light dance movements

Yi-Huang Su*

*** Correspondence:** Yi-Huang Su: yihuang.su@tum.de

# Table S1. Mean onset time asynchronies between the beat of leg movement and the closest beat of trunk movement, and the standard deviation of these asynchronies, for each stimulus condition in Experiment 2. The beat onset times of the leg and trunk movements in *Charleston* are both defined by the velocity cues, whereas the beat onset times of the *Balboa* leg movements are defined by the position cues (see result section of Experiment 2).

|  | *Charleston* 400 ms TM | *Charleston* 400 ms no TM | *Charleston* 450 ms TM | *Charleston* 450 ms no TM | *Balboa* 400 ms TM | *Balboa* 400 ms no TM | *Balboa* 450 ms TM | *Balboa* 450 ms no TM |
| --- | --- | --- | --- | --- | --- | --- | --- | --- |
| Mean onset asyn. (ms) | 148 | 129 | 116 | 143 | 106 | 118 | 136 | 129 |
| SD of onset asyn. | 41.7 | 56.6 | 22.9 | 53.8 | 45.3 | 61.2 | 62.1 | 55.8 |
